# Effectiveness of Fucoidan on Supplemental Therapy in Cancer Patients: A Systematic Review

**DOI:** 10.3390/healthcare10050923

**Published:** 2022-05-17

**Authors:** Chih-Jung Wu, Tzu-Pei Yeh, Ya-Jung Wang, Hsiao-Fang Hu, Shiow-Luan Tsay, Liang-Chih Liu

**Affiliations:** 1 Department of Nursing, HungKuang University, No. 1018, Section 6, Taiwan Boulevard, Shalu District, Taichung City 433304, Taiwan; elvaamy@gmail.com; 2 Department of Hematology and Oncology, China Medical University Hospital, No. 2, Yude Road, North District, Taichung City 404332, Taiwan; sharonhu5@gmail.com; 3 Department of Nursing, China Medical University, No. 100, Section 1, Jingmao Road, Beitun District, Taichung City 406040, Taiwan; tzupeiyeh@mail.cmu.edu.tw; 4 Department of Nursing, China Medical University Hospital, No. 2, Yude Road, North District, Taichung City 404332, Taiwan; 5 Department of Nursing, Da-Yeh University, No. 168, University Road, Dacun, Changhua 51591, Taiwan; wangyj@mail.dyu.edu.tw (Y.-J.W.); sltsay@mail.dyu.edu.tw (S.-L.T.); 6 Department of Breast Surgery, China Medical University Hospital, No. 2, Yude Road, North District, Taichung City 404332, Taiwan; 7 College of Medicine, China Medical University, No. 100, Section 1, Jingmao Road, Beitun District, Taichung City 406040, Taiwan

**Keywords:** fucoidan, survival time, disease progression status, anti-inflammatory effects, nutrition status, adverse effect, metastatic cancer patients

## Abstract

Purpose: Fucoidan is a dietary supplement which is commonly used by cancer patients. However, despite evidence of positive effects in cell culture environments, there are currently no clinical guidelines for supplementary use of fucoidan in cancer patients. This study aims to evaluate the effectiveness of fucoidan supplemental use. Methods: A systematic literature search was conducted using databases including Cochrane Library, JBI, PubMed, Embase, and CINAHL. All original studies on fucoidan for supplemental use in cancer patients were included. The search was made in databases without time restriction. The outcomes included disease progression status, inflammatory markers, nutritional status, adverse effects, and quality of life. The appraisal tool used was JBI-MAStARI. Results: Four studies were included: One randomized controlled trial and three quasi-experimental studies. Meta-analysis was not applied due to the heterogeneity of measurement tools. Overall sample size was 118. Most participants were metastatic colorectal and gastric cancer patients. Two studies revealed a significantly longer survival time and chemotherapy treatment periods with fucoidan use. Positive but insignificant effects of disease control rate, inflammatory markers, nutrition status, fatigue, and financial difficulty were shown in those using fucoidan. Conclusions: The results of this systematic review indicate that the effects of fucoidan were inconsistent with clinical outcomes in metastatic or recurrent cancer patients. Only four studies were included, and heterogeneity in methodologies and relatively small sample sizes limited the research consensus. Although cause and effect between fucoidan and the survival time, disease control or adverse effects could not be confirmed, this study includes the most research on fucoidan in humans.

## 1. Introduction

The use of complementary and alternative medicine (CAM) has steadily increased, particularly among cancer patients. CAM includes practices that are not typically part of conventional medical care, such as homeopathy, acupuncture, osteopathy, chiropractic, diet, herbal medicines, and use of biologic products [1,2]. Up to 90% of cancer patients reported consuming at least one type of CAM during their cancer treatment, and 70% of them did not discuss potential use of CAM with their physicians prior to beginning treatment [3]. The most common CAM that cancer patients use are vitamins and minerals, natural products, diet, massage, and herbs. Moreover, cancer patients spent US $52 billion on the consumption of CAM in the US [4]. Fucoidan is one popular natural dietary supplement for cancer therapy. In Taiwan, the use of fucoidan as a supplemental intervention has increased in cancer patients and in chronic disease patients during the last decade, and the annual production value of fucoidan- related products is more than US $100 million [5].

“Fucoidan” refers to a class of complex fucose-rich sulphated carbohydrate compounds extracted from various species of brown, green, and red marine macroalgaes or echinoderms [6]. The existence of fucoidan was confirmed in 1913 [7]. Fucoidan compounds principally consist of a α-1, 3-linked or α-1, 4-linked backbone, mainly with repeated L-fucose and sulfate groups, along with small proportions of D-galactose, D-xylose, D-mannose, and uronic acid. Variations of this basic structure are found in fucoidans from different kinds of brown seaweeds [8]. The absorption, metabolism, biological activity, and effect of fucoidan depend on the range of molecular weight, which vary depending on the methods of extraction and seaweeds used. In one method of categorization, fucoidan extracts up to 10 kilodalton (kDa) are called low molecular weight fucoidan (LMWF), from 10 to 10,000 kDa are called middle molecular weight fucoidan (MMWF), and more than 10,000 kDa are called high molecular weight fucoidan (HMWF) [6]. The influence of fucoidan on the activity and function of immune cells varies depending on molecular weight [9]. An extract including compounds ranging from 5–37 kDa has been shown to increase cellular production of anti-oxidation and cytotoxicity [10], while HMWF may enhance immune activity and prevent splenic cell necrosis [9]. Fucoidan has attracted widespread attention from cancer patients and their families because of its benefits, such as anti-oxidation, anti-cancer, anti-coagulation, anti-inflammatory, and anti-viral effects [11]. Therefore, fucoidan has become a broadly used supplemental therapy in many countries, such as Japan, Taiwan, and Australia [12,13].

In animal experiments, all studies indicated the anti-cancer effects of fucoidan, including anti-inflammation, anti-oxidation, anti-aging, lowering cholesterol, and stabilizing blood sugar [14,15,16,17]. However, fucoidan research in humans is sparse, and the completed studies were done in patients with cancer and hepatitis. In a randomized controlled trial (RCT) study, the result showed that fucoidan may decrease the blood alanine transaminase (ALT) level and maintain liver function in nonalcoholic fatty liver disease [15]. Among breast cancer patients who were taking hormone therapy and fucoidan at the same time, the pharmacokinetics were not influenced [12].

Many practitioners and cancer patients have shown interest in fucoidan products. Several investigations of fucoidan have been carried out in the past decade. However, most research was limited to in vivo or in vitro rather than clinical application and effects evaluation [18,19]. Two review studies have evaluated information on the effects and functions of fucoidan [18,19]. To date, no published review article has investigated the effectiveness of supplemental purpose of fucoidan in cancer patients. In addition, there are currently no clinical guidelines for supplementary use of fucoidan in cancer patients. Therefore, this systematic review (SR) aims to evaluate clinical use and effectiveness of fucoidan. The review results are necessary to establish recommendations of fucoidan use for cancer patients and healthcare providers.

## 2. Methods

### 2.1. Study Design and Search Strategy

In November 2020, a systematic search of literature was performed in Cochrane Library, Joanna Briggs Institute (JBI) library, PubMed, EMBASE, and CINAHL to identify original studies that evaluated the efficacy of fucoidan in patients receiving cancer therapy.

### 2.2. Eligibility, Selection, and Data Extraction

To select eligible articles for this review, population, intervention, comparators to the intervention, outcomes (PICO), and study designs were first defined as described below. The population was cancer patients who were more than 18 years old, and were undergoing cancer treatment. The intervention was using fucoidan as a complementary treatment, and the comparators could be none, usual care, or placebo. Outcomes included disease progression status, anti-inflammatory status, nutritional status, adverse effects, and quality of life. This review considered studies that focused on quantitative research, including intervention studies, controlled clinical trials, randomized controlled trials (RCT), and quasi-experimental studies. Studies published in English and Chinese were included in this review. Studies on fucoidan conducted in vivo, in vitro or in cells were excluded from this review. All studies were screened on the basis of title and abstract by two independent researchers (CJW and YJW). Data were extracted from papers using the standardized data extraction tool from JBI-MAStARI. The data extraction included specific details about the interventions, populations, study methods, and outcomes that were significant to answer the review questions.

### 2.3. Data Analysis

All results were subject to double data entry. The instruments for outcome measurement were different among studies, therefore meta-analysis was not conducted.

Outcomes were grouped into two categories: (1) Clinical outcomes, including overall survival, mean survival time, chemotherapy treatment periods, disease control status, anti-inflammatory, and prognostic nutritional indexes (PNIs); and (2) patient-reported outcomes, such as adverse effects and quality of life. The disease control status was defined as rates of complete response (CR), partial response (PR), and stable disease (SD). The PNIs was calculated following the serum albumin and total lymphocyte count.

### 2.4. Quality Assessment

The quality of each study was assessed using the JBI critical appraisal tools. The JBI MAStARI instrument was used for RCT and quasi-experimental studies (non-randomized experimental studies) [20]. The original JBI critical appraisal tool for RCT is a 13-question checklist with four options (“yes,” “no,” “unclear”, and “not/applicable”). The items of the RCT checklist included: (1) was true randomization used for assignment of participants to treatment groups; (2) was allocation to treatment groups concealed, treatment groups similar at the baseline; (3) were participants blind to treatment assignment, (4) were those delivering treatment blind to treatment assignment; (5) were outcomes assessors blind to treatment assignment; (6) were treatment groups treated identically other than the intervention of interest; (7) was follow-up completed and if not, were differences between groups in terms of their follow-up adequately described and analyzed; (8) were participants analyzed in the groups to which they were randomized; (9) were outcomes measured in the same way for treatment groups; (10) were outcomes measured in a reliable way; (11) was appropriate statistical analysis used; (12) was the trial design appropriate; and (13) were any deviations from the standard RCT design (individual randomization, parallel groups) accounted for in the conduct and analysis of the trial?

The original JBI critical appraisal tools for quasi-experimental studies had nine items with four options (“yes,” “no,” “unclear”, and “not/applicable”). The nine items included: (1) is it clear in the study what is the ‘cause’ and what is the ‘effect’ (for sample, there is no confusion about which variable comes first); (2) were the participants included in any comparisons similar; (3) were the participants included in any comparisons receiving similar treatment/care, other than the exposure or intervention of interest; (4) was there a control group; (5) were there multiple measurements of the outcome both pre and post the intervention/exposure; (6) was follow-up completed and if not, were differences between groups in terms of their follow-up adequately described and analyzed; (7) were the outcomes of participants included in any comparisons measured in the same way; (8) were outcomes measured in a reliable way; and (9) was appropriate statistical analysis used?

## 3. Results

### 3.1. Study Selection

**Figure 1:**
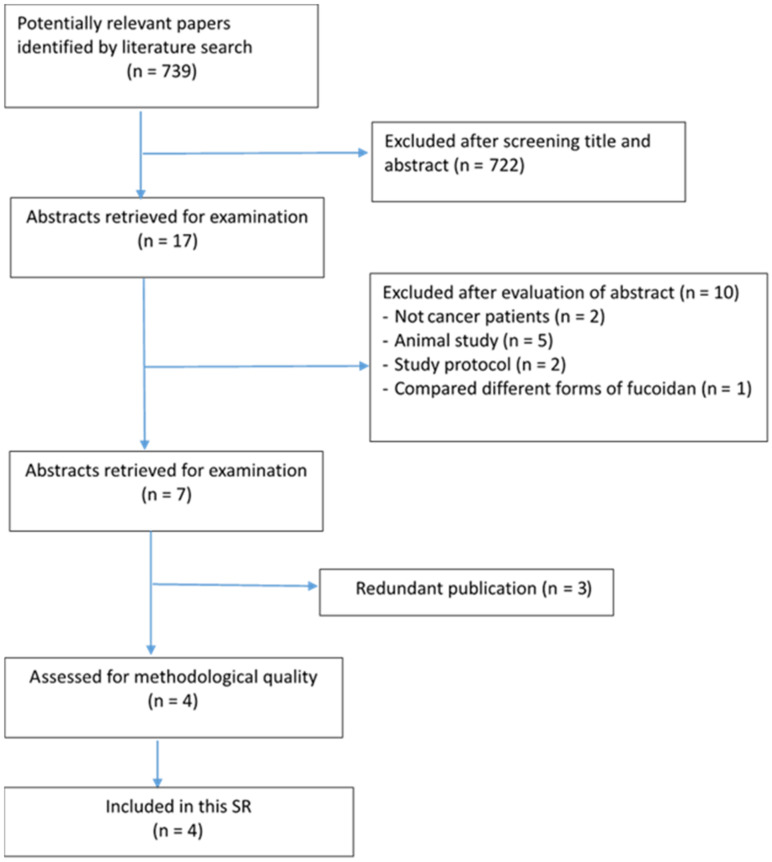
Flow chart for identification and selection of studies.

There were 739 potentially relevant papers identified by literature searching. Based on assessing the titles, 722 articles were excluded. Primary and secondary reviewers assessed the abstracts of the 17 remained studies, and finally identified four articles that met the inclusion criteria for this SR (Figure 1). The selected articles included one RCT and three quasi-experimental studies.

### 3.2. Characteristics of the Included Studies

Table 1 shows the characteristics of the included studies. The four studies were published from 2011 to 2018 and recruited patients with metastatic colorectal cancer or advanced gastric cancer in Japan and Taiwan [5,13,21,22]. The number of patients ranged from 20 [13] to 54 [5]. Three quasi-experimental studies were from Japan, and the RCT was from Taiwan. The fucoidan was taken 4–4.05 g/day of LMWF liquid form [13,21,22] or 4 g of LMWF (low molecular weight fucoidan) powder twice a day [5] for 1 to 6 months. All articles focused on patient-reported side effects, blood tests for toxicity analysis, and survival time; and two of them focused on quality of life.

**Table 1:** Detailed information of included studies.

Author (Year), Country	Participants	Fucoidan Source/Molecular Weight	Intervention vs. Control	Intervention Period	Outcome Measurements (also See Table 2)
Randomized controlled trials					
Tsai et al. [5] Taiwan	Metastatic Colorectal Cancer, n = 54 Median age: 57.46 ± 12.15(F) 62.38 ± 11.72(NF)	Sargassum hemiphyllum, LMF	4 g of LMF powder vs. cellulose powder	6 months	Clinical Patient reported
Quasi-experimental study					
Ikeguchi et al. [21] Japan	Advanced or recurrent colorectal cancer, n = 20 Mean age: 71.3 ± 7.5(F) 69.6 ± 8.8(NF)	Cladosiphon okamuranus, HMF	150 ml/day liquid (total 4.05 g fucoidan) versus no fucoidan	6 months	Clinical Patient reported
Ikeguchi et al. [22] Japan	Advanced gastric cancer, n = 24 Mean age: 61.2 ± 11(F) 63.3 ± 16.2(NF)	Cladosiphon okamuranus, HMF	150 mL/day liquid (total 4.05 g fucoidan) versus no fucoidan	6 months	
Takahashi et al. [13] Japan	Metastatic cancer n = 20 Mean age: 58.9 (Single group study)	Cladosiphon novae-caledoniae, LMF	400 mL/day liquid fucoidan (total 4 g fucoidan)	4 weeks	Clinical Patient reported

Note: F: With fucoidan; NF: No fucoidan. LMF: lowmolecular weight fucoidan; HMF: high molecular weight fucoidan.

**Table 2:** Important outcomes and results of included studies—clinical outcomes and patient-reported outcomes.

Variables	Reference	Results (Fucoidan Use Group vs. Control Group, or Fucoidan Only)
Clinical outcomes		
Disease progression status		
*Survival time (ST)*		
	Ikeguchi et al. [21]	8 (80%) vs. 6 (60%) patients still survived at 27th months, *p* = 0.314
	Tsai et al. [5]	18.04 vs. 12.96 months, *p* = 0.092
	Ikeguchi et al. [22]	Mean survival time; 12.0 vs. 8.0 months, *p* = 0.039
	Takahashi et al. [13]	Median survival time; 13.0 (IL-1β level decreased) vs. 5.0 months (IL-1β level not decreased), *p* = 0.02 (Single group study)
*Progression-free survival (PFS)*		
	Tsai et al. [5]	15.93 vs. 10.80 months, *p* = 0.075
*Overall response rate (ORR)*		
	Tsai et al. [5]	60.7% vs. 46.2%, *p* = 0.284
*Disease control rate (DCR)*		
	Tsai et al. [5]	92.8% vs. 69.2%, *p* = 0.026
*Chemotherapy treatment periods*		
	Ikeguchi et al. [22]	7.4 vs. 4.6 months, *p* = 0.004
	Ikeguchi et al. [21]	19.9 vs. 10.8 cycles, *p* = 0.016
Anti-inflammatory change over time		
	Takahashi et al. [13] (Single group study)	1.IL-1B (358.2 → 189.9, *p* = 0.01) 2.IL-6 (2198.6 → 1522.8, *p* = 0.02) 3.TNF-a (4819.4 → 3257.2, *p* = 0.03)
Prognostic nutritional indexes (PNIs)		
	Ikeguchi et al. [22]	47.6 vs. 39.4, *p* = 0.028
Patient-reported outcomes Reference Results (fucoidan vs. control, or fucoidan change overtime)		
Quality of life (QoL)		
	Takahashi et al. [13]	No significant difference over time (QoL score 58.3 ± 5.3 → 58.3 ± 4.8; *p* = 0.92) (Single group study) (QoL tool: EORTC QLQ-C30)
	Tsai et al. [5]	No significant difference (QoL tool: EORTC QLQ-CR29)
Adverse effects (AEs)		
	Takahashi et al. [13]	Financial difficulty score reduced (QoL score 35.0 ± 7.0 → 20.0 ± 5.6; *p* < 0.01) (AEs tool: EORTC QLQ-C30)
	Ikeguchi et al. [21]	General fatigue (Incidence 10% vs. 60%, *p* = 0.019) (AEs tool: NCI CTCAE)
	Tsai et al. [5]	Oral mucositis (Incidence 50% vs. 65.4%, *p* = 0.253) Pruritus (Incidence 35.7% vs. 53.9%, *p* = 0.180) Vomiting (Incidence 35.7% vs. 53.9%, *p* = 0.180) Taste problem (Incidence 64.3% vs. 80.8%, *p* = 0.177) Bloody stool (Incidence 14.3% vs. 30.8%, *p* = 0.145) (AEs tool: NCI CTCAE and EORTC-QLQ-CR29)
	Ikeguchi et al. [22]	Fatigue (3 vs. 7 patients, *p* = 0.098) Diarrhea (0 vs. 3 patients, *p* = 0.064) (AEs tool: NCI CTCAE)

Note: IL-1B: interleukin 1, beta; IL-6: interleukin 6; TNF-a: tumor necrotic factor-alpha. EORTC QLQ-C30: European Organization for Research and Treatment of Cancer Quality of Life Questionnaire C30. NCI CTCAE: National Cancer Institute Common Terminology Criteria for Adverse Events.

### 3.3. Methodological Quality of Included Studies

There was one RCT study which met the strict standards of RCT design. In this study, a double-blind design was adopted to avoid the bias from demand characteristics or the placebo effect. Fifty-four patients were randomly assigned to receive either fucoidan or placebo groups [5]. Three quasi-experimental studies included a single group pre-and-post evaluation study [13] and two studies with intervention and control groups but without random allocation [21,22]. All studies presented in reliable outcome measurements and applied appropriate statistical analysis methods.

The methodological quality of included studies ranged from medium to high, scored using the JBI-MAStARI critical appraisal checklist presented in Appendix A.

### 3.4. Clinical Outcomes

All studies reported clinical outcomes as details presented in Table 2, including disease progression status, inflammatory markers, and prognostic nutritional indexes (PNIs).

#### 3.4.1. Disease Progression Status

Disease progression statuses were evaluated from the overall or mean survival time, disease control status, and chemotherapy treatment periods. One study revealed that the mean survival time was significantly longer than 4 months, in the fucoidan treatment group than in the no fucoidan treatment group [22]. Another study specifically mentioned that patients showed less interleukin 1-beta (IL-1B) in the fucoidan group and had significantly longer median survival time, by 8 more months [13]. 

Only one study reported results of radiology that divided DCR into complete response (CR) plus stable disease (SD), and progressive disease (PD). The second outcomes, which included progression-free survival (PFS), overall response rate (ORR), and disease control rate (DCR), indicated that DCR was significantly higher in the fucoidan group than in the control group (92.8% vs. 69.2%, *p* = 0.026), and the fucoidan group also had a longer median follow-up period of 11.5 months5. Two studies reported significantly longer chemotherapy treatment periods (7.4 months, *p* = 0.004) [22] and higher average number of treatment cycles (19.9 cycles, *p* = 0.016) [21] in fucoidan groups than no fucoidan groups.

#### 3.4.2. Anti-Inflammatory Effects

Takahashi indicated that patients showed decreased IL-1B after fucoidan administration for two weeks and had a significantly longer survival time compared with IL-1B non-responders (median survival time = 13.0 vs. 5.0 months; *p* = 0.02) [13]. The results showed that the values of three main pro-inflammatory cytokines, including IL-1B (358.2 ± 62.7 → 189.9 ± 32.0, *p* = 0.01), IL-6 (2198.6 ± 564.3 → 1522.8 ± 367.0, *p* = 0.02), and TNF-a (4819.4 ± 772.0 → 3257.2 ± 648.6, *p* = 0.03), were significantly reduced after administration of fucoidan for two weeks.

#### 3.4.3. Prognostic Nutritional Indexes (PNIs)

The PNI value was significantly greater in the fucoidan group than control group (47.6 ± 6.1 vs. 39.4 ± 8.2, *p* = 0.028) post treatment [22]. This indicated that cancer patients in the fucoidan group had significantly better nutrition profiles compared to their counterparts.

### 3.5. Patient-Reported Outcomes

All studies also measured patient-reported outcomes including adverse effects and quality of life (Table 2).

#### 3.5.1. Adverse Effects (AEs)

All studies applied the National Cancer Institute Common Toxicity Criteria (NCI CTC) to evaluate AEs. One study reported the financial difficulty score as the AE measure and found it was significantly improved after administration of fucoidan for four weeks (35.0 ± 7.0 → 20.0 ± 5.6, *p* < 0.01) [12]. Moreover, patient-reported general fatigue was significantly reduced in the fucoidan groups compared with the control group [21]. However, comparisons in AEs showed no significant difference in two studies, although participants in no fucoidan groups showed higher percentages of oral mucositis, pruritus, vomiting, taste problems, bloody stool, fatigue, and diarrhea [5,22]. 

#### 3.5.2. Quality of Life 

One study used the European Organization for Research and Treatment of Cancer Quality of Life Questionnaire Core 30 (EORTC QLQ-C30) [13], and one study used the European Organization for Research and Treatment of Cancer Quality of Life Questionnaire Core 29 (EORTC QLQ-CR29) [5] to examine changes in quality of life. However, neither study found significant changes of quality of life the two groups [5,13].

## 4. Discussion

This SR found that the outcomes in this review indicated no poorer results or harmful effects in participants who used fucoidan. Importantly, fucoidan had positive effects as a supplemental treatment on better disease progression status (such as survival time, disease control rate, chemotherapy treatment periods), good anti-inflammatory status, PNIs, and less AEs (financial difficulty and fatigue). However, the heterogeneity in the research contexts and methodologies leaded conservative conclusion of recommendation in fucoidan use. 

### 4.1. Disease Progression Status

The survival time is an important index of cancer treatment outcomes [23]. Results of this SR indicated that participants in fucoidan groups had significantly prolonged survival time. One study using a one group pre- and post-test study design showed significantly prolonged medium survival time in participants whose IL-1β level decreased [13], and the other quasi-experimental study showed significantly prolonged mean survival time in fucoidan use group [22]. According to the survival rate reports [24], many research studies applied either the mean survival time or the medium survival time according to the scale of study sample size. Both studies may offer valuable information on measuring outcomes in new drugs or CAM intervention in clinical settings. However, in this SR, one study with quasi-experimental study and one with single group study design in small sample sizes, the result of survival time was treated with reserve.

Moreover, the studies evaluated in this SR only included metastatic and recurrent gastro-intestinal cancer patients, and this outcome may not be generalizable to patients of other types of cancer. A literature review on patient’s needs indicated that the common unmet needs for advanced cancer patients were emotional support, dealing with fatigue, and being informed about benefits as well as side effects of treatment [25], and the use of fucoidan may improve survivor’s dealing with fatigue and side-effects of treatment. Therefore, the use of fucoidan may be clinically complementary to more traditional treatments. The DCR mainly relied on image examination and response evaluation criteria in solid tumors, which is an objective assessment guideline [26] Evidence suggests that PFS, ORR, and DCR are crucial indices for evaluating cancer treatment response and the effect of drug activity against cancer. However, the PFS is limited because of the variation of inter-rater reliability [27,28], and only one study in this SR included the above-mentioned indices as outcome measurements [5]. Nevertheless, only the DCR outcome was significant in this RCT study, despite meeting the RCT appraisal requirements. However, the results remained conservative for clinical application due to the small sample sizes in these selected studies. 

#### 4.1.1. Inflammatory Markers

Fucoidan was approved to be an anti-inflammatory substance in vitro and in vivo [29]. In this SR, only Takahashi found significant reductions in inflammatory markers in the fucoidan group [13]. Literature suggested that chemotherapy-induced symptoms were associated with inflammatory markers. For instance, fatigue was related to IL-1B, IL-6, and tumor necrosis factor-alpha (TNF-a) [30]. Therefore, fucoidan might lessen inflammatory reactions, which may also reduce side effects of chemotherapy.

#### 4.1.2. Adverse Effects

In this SR, one study found significantly reduced fatigue [21] and another suggested significantly reduced financial difficulty [13] in fucoidan groups. Two studies found that the AE incidences of AEs, such as athrombocytopenia, peripheral neuropathy, liver dysfunction, oral mucositis, pruritus, vomiting, taste problems, and bloody stool, were reduced in those who used fucoidan; however, the AE reduction was not statistically significant [5,21]. The possible reasons for this lack of significance may because of the small sample sizes and the heterogeneity of the samples. For example, Ikeguchi et al. recruited only 20 participants but included six types of cancer [21]. Tsai et al. included only metastatic colorectal cancer patients; therefore, the sample was homogeneous and the observation time period of AEs was not reported [5]. Moreover, this study only reported the difference of incidence without comparing the severities of AEs between fucoidan and the control group; it might fail to determine the real difference between groups.

Financial difficulty was significantly improved after four weeks of the fucoidan use [13]. This result was different from some studies of CAM utilization, which concluded that financial difficulty worsened in those who used [31,32]. This might be because the studies in this SR were mainly sponsored by research institutes who are the sellers of commercial fucoidan, while the CAM in other studies represented an extra expense to participants [31,32].

Fucoidan products are expensive, but studies selected in this SR did not investigate the cost-effectiveness of fucoidan use. If cancer patients would like to use fucoidan during chemotherapy, it would cost about 10 USD/day in Taiwan. The use of fucoidan would be expected to last for a long time for cancer patients. Therefore, fucoidan would be only recommended for those cancer patients who can afford it.

#### 4.1.3. Quality of Life

Quality of life did not show significant improvement in fucoidan groups. This result was aligned with research of CAM in various countries, such as Malaysia, Ethiopia, and Korea [31,32,33]. It may because the quality of life does not change over short periods, and the disease characteristics as well as treatment plans of advanced cancers were so different. From the literature, we may conclude that common reasons for using CAM in advanced cancer patients were improving immune functions, maintaining physical energy, and psychological well-being; these expected factors are usually related to impact quality of life [34,35].

### 4.2. Strengths and Limitations

This is the first SR to examine the effectiveness of fucoidan in metastatic or recurrent cancer patients. The results indicate that there are positive effects of fucoidan on disease progression status, inflammatory markers, nutritional status, and fatigue. Some limitations of this review need to be addressed. Firstly, only four articles focused on the effects of fucoidan, and only one of them was an RCT study. Second, outcome measurement tools varied widely among studies, which largely increased the difficulties in integrating and interpreting those research results together. Third, the results of individual outcome in different studies were inconsistent, and the sample populations were mainly limited to metastatic and recurrent gastro-intestinal cancer patients. Therefore, it remains unclear whether the effects of fucoidan in other types of cancer would be similar. 

### 4.3. Implications for Clinical Practice

Results of this SR indicate that patients of metastatic or recurrent gastric and colorectal cancer who used fucoidan as a complementary treatment had significantly prolonged survival time, better anti-inflammatory profiles, and reduced fatigue. However, only four studies were included in this SR, the overall sample size was relatively small, and the participants were advanced gastro-intestinal cancer patients. Therefore, fucoidan might be recommended for metastatic or recurrent gastro-intestinal cancer patients, if their financial status allows. However, further RCT studies with larger sample sizes in this area are needed in order to confirm the effects of fucoidan in different diseases and obtain generalizable results. Meanwhile, the sampling homogeneity with a single type of cancer should be noted. Although this review paper indicated the limitations of research population as participants were recruited from metastatic or recurrent cancer patients with gastro-intestinal cancers, fucoidan could still be a beneficial supplement for patients with various diseases due to its multiple mechanisms in disease treatments [14,15,16,17].

### 4.4. Suggestions for Future Research

In further research in the future, in addition to enrolling a larger sample size, research in different cancer types and stages and even in various diseases should be considered; this may increase the evidence of fucoidan in humans, and its effects or benefits to clinical patients could be further explored and confirmed.

## 5. Conclusions

Fucoidan may have positive benefits on survival time, disease control rate, reducing side effects during chemotherapy treatment periods, lowering inflammatory markers, and minimizing in patients’ fatigue, whether in patients with cancer metastatic or recurrent. Fucoidan should be recommended for metastatic or recurrent gastric or colorectal cancer patients who can afford the expense. Further studies should be done to confirm the effects of fucoidan on cancer patients in longitudinal research design, ideally recruiting patients with varied cancer types or patients with other diseases; thus, the use of fucoidan could be more comprehensively evaluated.

## Data Availability

All of the included literatures could be found in online databases.

## Appendix A

**Table A1:** Appraisal result of study quality for quasi-experimental studies using the JBI-MAStARI (see Method Section 2.4 for questions 1–9).

Citation	Q1	Q2	Q3	Q4	Q5	Q6	Q7	Q8	Q9	Score
Ikeguchi et al. [21]	Y	Y	N/A	Y	Y	Y	Y	Y	Y	8
Takahashi et al. [13]	Y	U	U	U	Y	Y	Y	Y	Y	7
Ikeguchi et al. [22]	Y	Y	N/A	Y	Y	Y	Y	Y	Y	8

Y = yes, N = no, U = unclear, N/A = not applicable.

**Table A2:** Appraisal result of study quality for the RCT using the JBI-MAStARI (see Method Section 2.4 for questions 1–13).

Citation	Q1	Q2	Q3	Q4	Q5	Q6	Q7	Q8	Q9	Q10	Q11	Q12	Q13	Score
Tsai et al. [5]	Y	Y	Y	Y	Y	U	U	Y	Y	Y	Y	Y	U	10

Y = yes, N = no, U = unclear, N/A = not applicable.
